# Nitric oxide-dependent immunosuppressive function of thymus-derived mesenchymal stromal/stem cells

**DOI:** 10.1186/s13062-023-00415-4

**Published:** 2023-09-18

**Authors:** Xiao Su, Xiaolei Li, Shiqing Wang, Xiaotong Xue, Rui Liu, Xiaojing Bai, Pixia Gong, Chao Feng, Lijuan Cao, Tingting Wang, Yayun Ding, Junjie Jiang, Yongjing Chen, Yufang Shi, Changshun Shao

**Affiliations:** First Affiliated Hospital of Soochow University, State Key Laboratory of Radiation Medicine and Protection, Institutes for Translational Medicine, Soochow University Suzhou Medical College, Suzhou, Jiangsu 215123 China

**Keywords:** Thymus, Mesenchymal stromal/stem cells, Immunomodulation, NO, Nestin

## Abstract

**Background:**

The thymus is required for T cell development and the formation of the adaptive immunity. Stromal cells, which include thymic epithelial cells (TECs) and mesenchymal stromal cells (MSCs), are essential for thymic function. However, the immunomodulatory function of thymus-derived MSCs (T-MSCs) has not been fully explored.

**Methods:**

MSCs were isolated from mouse thymus and their general characteristics including surface markers and multi-differentiation potential were characterized. The immunomodulatory function of T-MSCs stimulated by IFN-γ and TNF-α was evaluated in vitro and in vivo. Furthermore, the spatial distribution of MSCs in the thymus was interrogated by using tdTomato-flox mice corssed to various MSC lineage Cre recombinase lines.

**Results:**

A subset of T-MSCs express Nestin, and are mainly distributed in the thymic medulla region and cortical-medulla junction, but not in the capsule. The Nestin-positive T-MSCs exhibit typical immunophenotypic characteristics and differentiation potential. Additionally, when stimulated with IFN-γ and TNF-α, they can inhibit activated T lymphocytes as efficiently as BM-MSCs, and this function is dependent on the production of nitric oxide (NO). Additionally, the T-MSCs exhibit a remarkable therapeutic efficacy in acute liver injury and inflammatory bowel disease (IBD).

**Conclusions:**

Nestin-positive MSCs are mainly distributed in medulla and cortical-medulla junction in thymus and possess immunosuppressive ability upon stimulation by inflammatory cytokines. The findings have implications in understanding the physiological function of MSCs in thymus.

**Supplementary Information:**

The online version contains supplementary material available at 10.1186/s13062-023-00415-4.

## Background

Mesenchymal stromal cells (MSCs), originally isolated from bone marrow (BM) [1], were fibroblast-like in morphology, adherent and nonhematopoietic stem cells. The MSCs in BM maintain the dynamic bone marrow hematopoietic microenvironment through supporting the differentiation and proliferation of hematopoietic stem cells (HSCs) [2, 3]. They have robust self-renewing ability and can differentiate into chondrocytes, osteoblasts and adipocytes [4]. In recent years, MSCs have attracted increasing attention due to their astonishing ability to coordinate tissue regeneration and regulate immune response in pre-clinical and clinical settings [5–8]. For instance, MSCs inhibit the overactivation of the immune system via the secretion of several immunosuppressive factors [9, 10], and are promising as a non-hormone therapy alternative for autoimmune diseases.

MSCs are highly heterogeneous, and some subpopulations can be identified by the expression of Nestin [3], Leptin receptor (LEPR) [11], Neuron-glia antigen2 (NG2) [12], and Pairing the associated homeobox protein 1 (PRX1) [13], among others. Different MSC subsets function distinctly. For instance, the Nestin^+^ MSCs in bone marrow contribute to the maintenance of HSCs [3]. NG2^+^ Nestin^high^ MSCs maintain a niche for quiescent HSCs, whereas LEPR^+^ Nestin^low^ MSCs provide a vital niche for activated HSCs [12]. Therefore, the specific characteristics and functions of MSCs in different tissues require further research.

MSCs are well-documented to have immunomodulatory function via regulating the differentiation and function of T lymphocytes [8]. T cell development occurs in the thymus [14–16], and is regulated by thymic epithelial cells (TECs) that secrete various growth factors and cytokines [17–20]. In addition, thymic mesenchymal cells (MCs), the non-TEC stromal cells, are predominantly found in the medulla and capsule [21]. In the medulla, lymphotoxin secreted by single positive thymocytes binds to the lymphotoxin β receptor (LTβR) expressed on medullary MCs, which is essential for their functional maturation. Mature MCs can induce T cell tolerance [22–25]. MCs produce fibroblast growth factor-7 (FGF-7), FGF-10, insulin-like growth factor-1 (IGF-1), IGF-2 and retinoic acid [26–30] to regulate the proliferation of TECs. Moreover, MCs are indispensable for the of maintenance of TECs and thymus regeneration [31, 32]. RNA-seq analysis of Lin^−^ 7-AAD^−^ Sca-1^+^ cells isolated from the thymus showed that thymic MCs express genes implicated in chemotaxis, efferocytosis and M2 polarization of macrophages, and resolution of inflammation, thus presumably maintaining a non-inflammatory environment [33]. Besides, thymic MCs express genes involved in mesenchymal–epithelial cross-talk and epithelial cell adhesion to basement membrane [33].

Nestin, an intermediate filament protein, is expressed in the early stages of development and was originally discovered in neuroepithelial stem cells [34]. Nestin is also expressed in some adult stem/progenitor cell populations and is frequently utilized as a marker of neural stem cells and MSCs [35–37]. Nestin-postive MSCs have been identified in lung, Peyer’s patches in intestine and spleen. However, their presence and distribution in thymus remain to be determined.

In this study, we aim to determine the spatial distribution of the mesenchymal stromal cells in mouse thymus (T-MSCs) and to characterize their immunomodulatory function. We found that Nestin + T-MSCs were mainly distributed in the medulla and the cortical-medulla junction. We further demonstrated that T-MSCs possess immunomodulatory property that is comparable to that of BM-derived MSCs.

## Results

### Isolation and characterization of thymus-derived MSCs (T-MSCs)

**Fig. 1:**
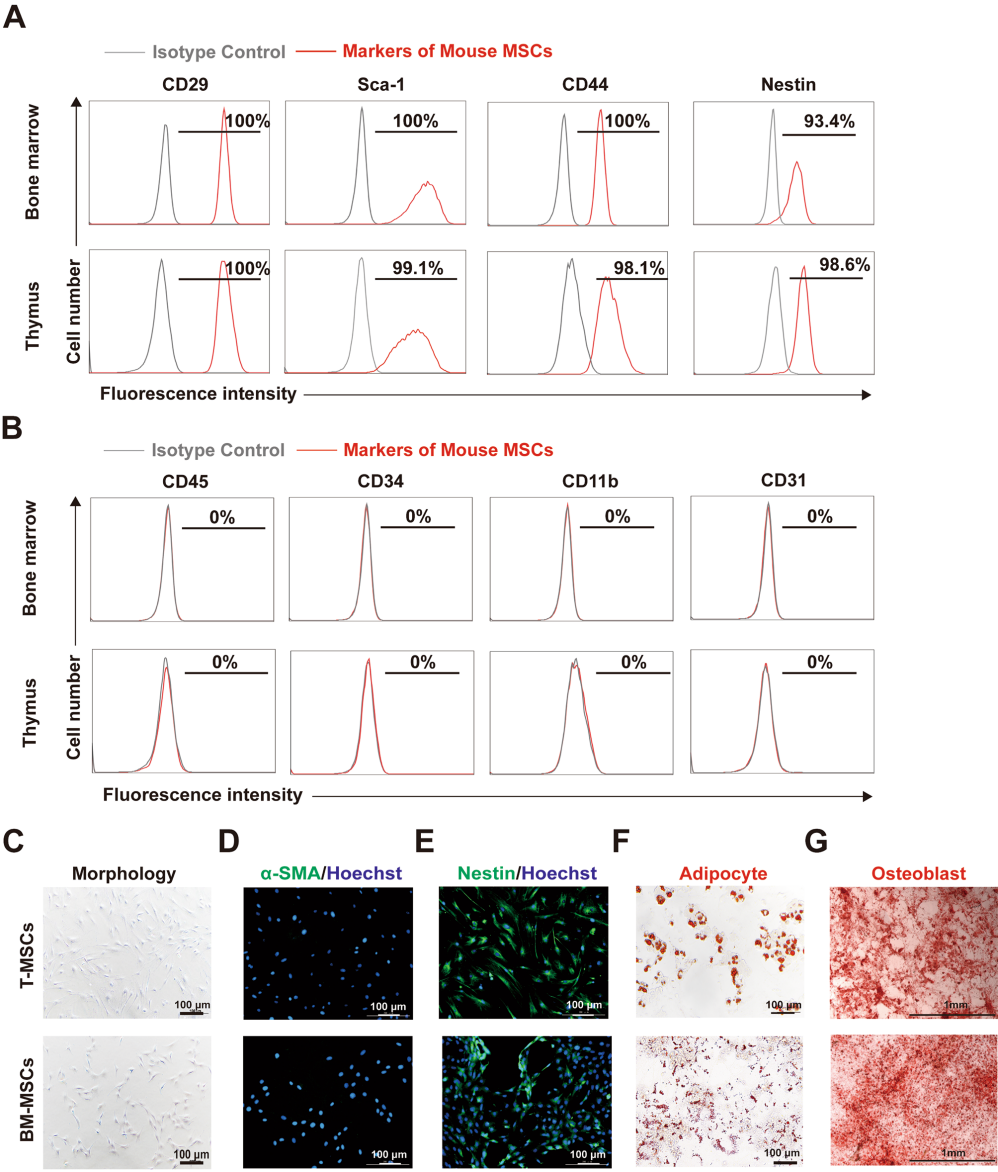
Multipotent differentiation of MSCs isolated from murine thymic tissue and bone marrow. **(A, B)** Expression of cell surface markers on T-MSCs and BM-MSCs as determined by flow cytometry. **(C-E)** Morphology was detected after fixation and analyzed with inverted microscope. α-SMA and Nestin expression was determined after fixation and staining with an Alexa Fluor® 488-coupled antibody against α-SMA and Nestin, respectively, nuclei were counterstained with Hoechst 33,342, and then analyzed under fluorescence microscope. Scale bars, 100 μm. **(F, G)** Adipogenic and osteogenic differentiation of T-MSCs and BM-MSCs was evidenced by Oil Red O-stained fat vacuoles or Alizarin Red S-stained calcium deposits, respectively. Scale bars, 100 μm or 1 mm

We isolated and propagated plastic adherent stromal cells from the bone marrow (BM) and thymus of 4–8 weeks C57BL/6 mice. The adherent cells were morphologically and immunophenotypically heterogeneous until passage 6 (data not shown). Therefore, adherent cells between passages 9 and 23 (T-MSCs, 9–14 passages; BM-MSCs, 9–23 passages) were used in all subsequent experiments. From passage 9 onwards, all stromal cell population was positive for expression of CD29, Sca-1, CD44 and Nestin (Fig. 1A). Flow cytometry analysis also demonstrated that MSCs are negative for CD45, CD31, CD34 and CD11b, the surface markers on hematopoietic cells (Fig. 1B). Overall, these results confirmed the previously reported presence of MSCs in the mouse thymus.

The T-MSCs were morphologically similar to BM-MSCs after 9 passages (Fig. 1C). Immunofluorescence staining indicates lack of expression of a-SMA, a marker for myofibroblasts, on BM- and T-MSCs, but confirmed the presence of Nestin in T-MSCs and BM-MSCs (Fig. 1D, E).

The MSCs are characterized by their ability to differentiate into several lineages, including osteoblastic and adipocytic lineages [38]. The T-MSCs were cultured under various conditions to assess their capacity to differentiate into committed lineages. Like BM-MSCs, T-MSCs acquired intracellular lipid droplets or deposited a calcium-rich mineralized matrix in adipogenic and osteogenic media (Fig. 1F, G), respectively, demonstrating the multi-differentiation potential of these cells.

We next compared T-MSCs, BM-MSCs and adipose-derived mesenchymal stromal/stem cells (ADSCs) in expression of representative genes by real-time PCR [9, 33, 39, 40] and found that the T-MSCs was more similar to BM-MSCs than to ADSCs (Supplementary Fig. 1).

### Nestin^+^ mesenchyml stromal cells are mainly distributed in the medulla and the cortical-medulla junction

**Fig. 2:**
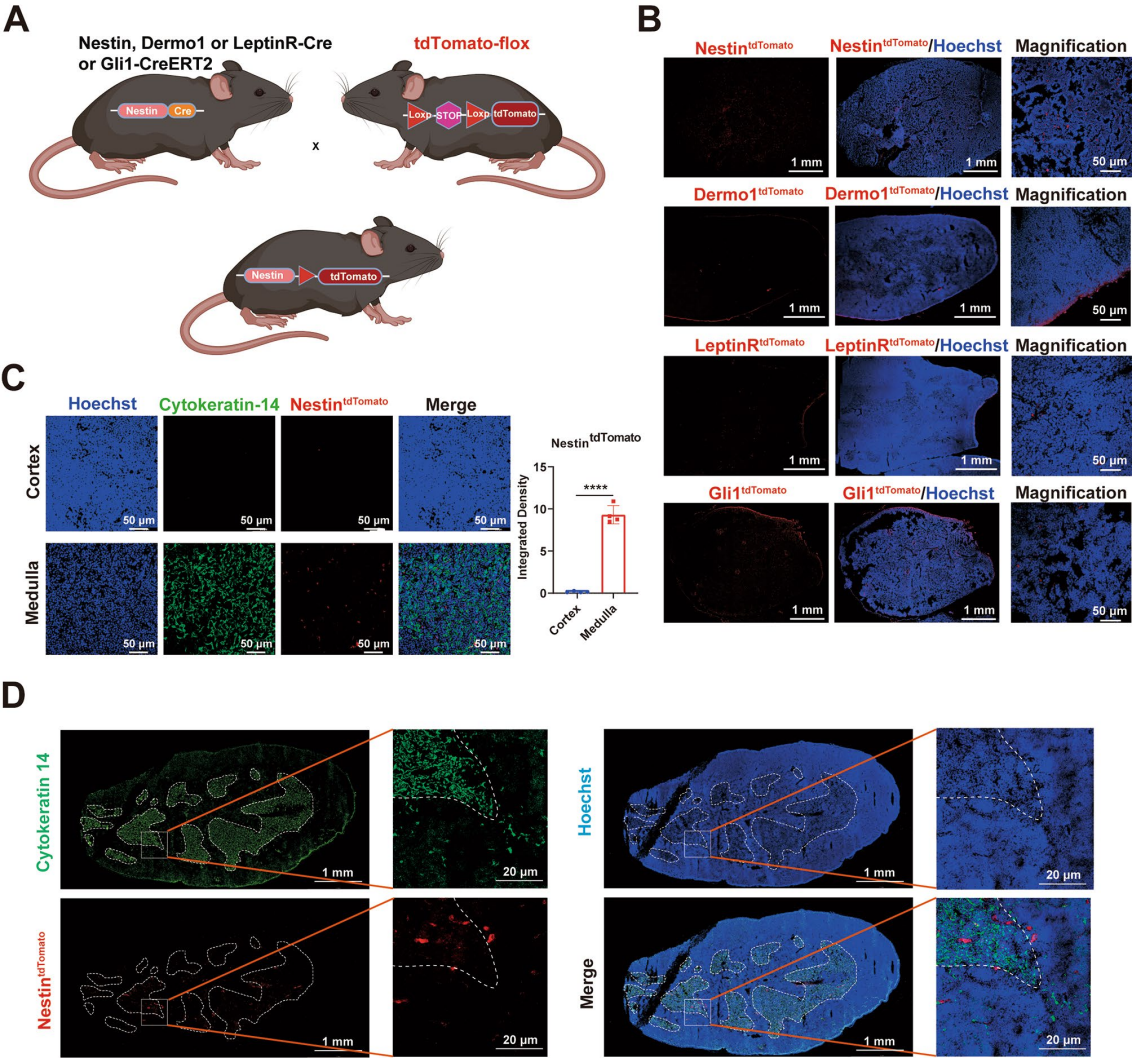
Distribution of Nestin, Dermo1, LeptinR or Gli1-expressing cells in the thymus. **(A)** Generation of Nestin, Dermo1, LeptinR or Gli1 fluorescent reporter mice. Nestin-Cre, Dermo1-Cre, LeptinR-Cre and Gli-CreERT2 mice were crossed to tdTomato-flox. **(B)** Nestin, Dermo1, LeptinR or Gli1 (Red) expression respectively in the thymus of Nestin-tdTomato, Dermo1-tdTomato, LeptinR-tdTomato or Gli1-tdTomato transgenic mice (n = 3). Representative immunofluorescence of thymus sections stained for nuclei (Hoechst, blue). **(C, D)** Nestin (Red) expression in the thymus of Nestin-tdTomato transgenic mice (C, D, n = 3). Representative immunofluorescence of thymus sections stained for Cytokeratin (green) and nuclei (Hoechst, blue). Scale bars, 20 μm, 50 μm and 1 mm. Data are represented as mean ± SEM. ****p < 0.0001

We next interrogated the distribution of MSCs in the thymus. Nestin^+^, Dermo1^+^, LeptinR^+^ or Gli1^+^ mesenchymal cells were reported to identify several subtypes of MSCs [8]. We crossed Nestin-Cre, Dermo1-Cre, LeptinR-Cre and Gli1-CreERT2 mice to tdTomato-flox mice, respectively, to locate the Nestin^tdTomato^, Dermo1^tdTomato^, LeptinR^tdTomato^ and Gli1^tdTomato^ cells in thymus (Fig. 2A). As shown in Fig. 2B, while Dermo1^tdTomato^, LeptinR^tdTomato^ and Gli1^tdTomato^ could all label capsule of the thymus, as well as various interior regions, the Nestin^tdTomato^ labeled cells were only detected in interior. Additionally, Nestin^+^ cells in the thymus were shown to express Sca-1^+^, but were CD45^−^ and Lin^−^ by flow cytometry analysis (Data not shown). These results suggest that Nestin^+^ cells derived from thymus likely represent a subset of mesenchymal stromal cells in the thymus [33, 41]. Using cytokeratin 14 as a specific marker of medulla thymic epithelial cells (mTECs) that distinguishes cortical and medullary regions of thymus, we observed that Nestin^tdTomato^ cells were mainly distributed in medulla and the cortical-medulla junction(Fig. 2C, D).

### T-MSCs possess immunosuppressive function

**Fig. 3:**
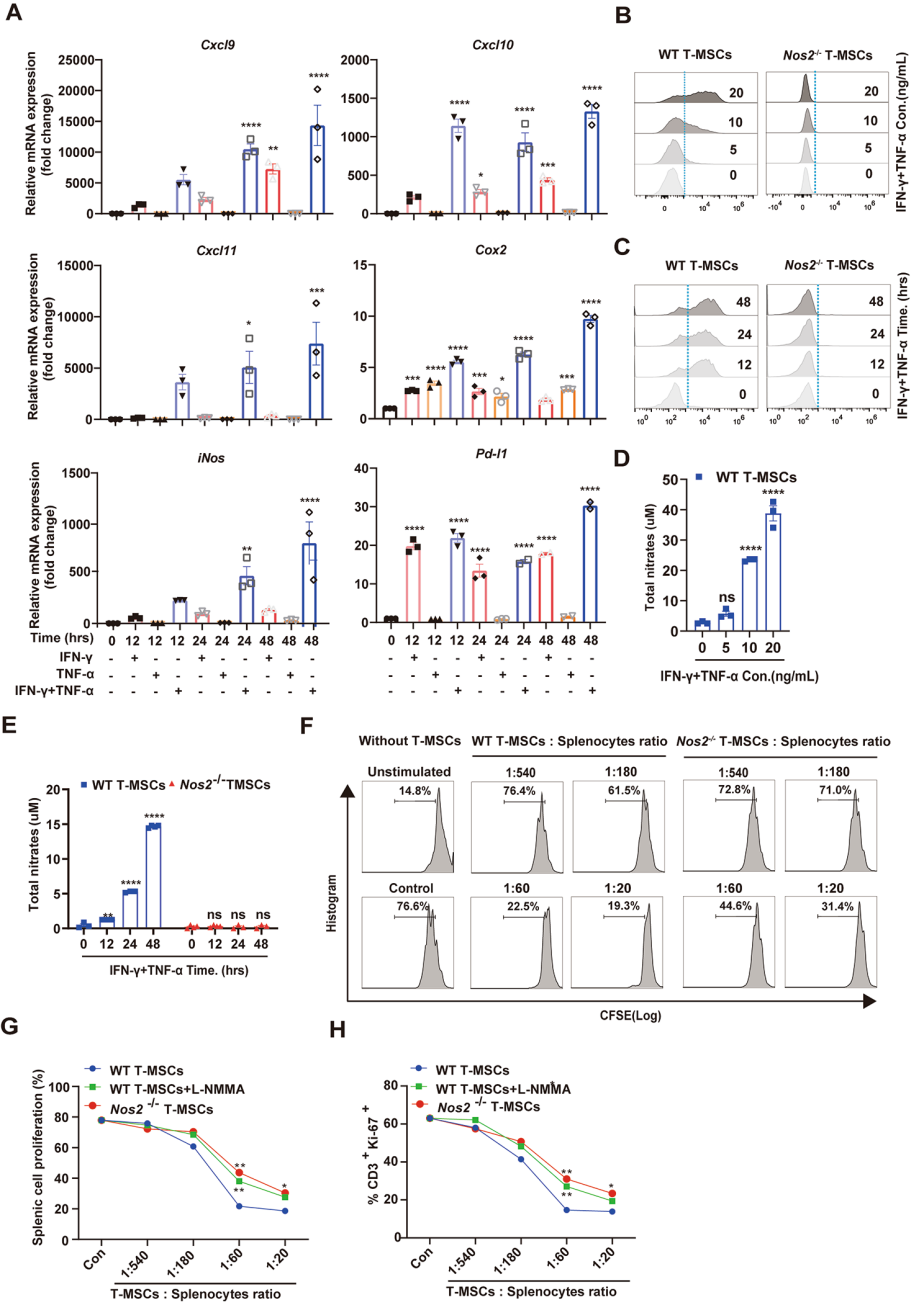
The immunosuppressive effect of T-MSCs on T cell proliferation is partially dependent on NO. **(A)** The expression levels of *Cxcl9, Cxcl10, Cxcl11, iNos, Cox2* and *Pd-l1* were detected by real-time PCR at 12 h, 24 h and 48 h after MSCs were stimulated with 20 ng/mL IFN-γ and TNF-α alone or synergically. **(B)** The expression of iNOS in T-MSCs was detected by flow cytometry after 24 h synergistic stimulation with different concentrations of IFN-γ and TNF-α. **(C)** The expression of iNOS in T-MSCs was detected by flow cytometry after different time synergistic stimulation with IFN-γ and TNF-α (20 ng/mL each). **(D)** Cell culture supernatant was obtained from different treatment groups in Fig. 3B, total nitrates level was assayed by a modified Griess reagent. **(E)** Cell culture supernatant was obtained from different treatment groups in Fig. 3C, total nitrates level was assayed by a modified Griess reagent. **(F)** T-MSCs and CFSE-labeled splenocytes were co-cultured for 48 to 72 h at the ratio of 1:20, 1:60, 1:180 and 1:540, respectively (anti-CD3/CD28 (1 µg/mL each) was added into the medium to stimulate the proliferation of splenocytes), and then fluorescence intensity of CFSE was detected by flow cytometry. **(G)** T-MSCs from *Nos2*^−/−^ or WT C57BL/6 mice were cocultured with fresh C57BL/6 splenocytes plus anti-CD3/CD28, with or without the iNOS inhibitor, L-NMMA (1 mM). Cell proliferation was assayed after 48 to 72 h. **(H)** The groups was consistent with Fig. 3G (expect for the splenocytes were not stained with CFSE), and after 48 h of culture, CD3 and Ki-67 were stained, then the proportion of CD3^+^Ki-67^+^ was detected by flow cytometry. Data are represented as mean ± SEM. ns, no significant difference, *p < 0.05, **p < 0.01, ***p < 0.001, ****p < 0.0001

Next, we analyzed the immune regulatory function of the Nestin^+^ cells in vitro. It is well documented that under the stimulation of proinflammatory factors, BM-MSCs exert immunosuppressive function by expressing effector molecules and chemokines [8]. Therefore, we investigated whether T-MSCs have similar functions. As shown in Fig. 3A, the expression of T cell-specific chemokines C-X-C motif chemokine ligand 9 (*Cxcl9*), *Cxcl10*, *Cxcl11* and immunosuppressive effector molecules *iNos*, *Cox2*, *Pd-l1* were significantly upregulated under the stimulation by proinflammatory factors (IFN-γ and TNF-α), and remained at a high level after 48 h, which was similar to what was observed in BM-MSCs (Supplementary Fig. 2A-F). These results suggest that IFN-γ and TNF-α can induce T-MSCs to produce immunosuppressive molecules.

BM-MSCs stimulated by proinflammatory cytokines produce high levels of chemokines, which, in turn, promote T cell chemotaxis [9]. Meanwhile, BM-MSCs expressed high level of adhesion molecules under proinflammatory cytokines and maintained contact with T cells [39]. Therefore, we tested whether T-MSCs respond similarly to the inflammatory cytokines. Indeed, splenic cells were predominantly localized in the close vicinity of IFN-γ/TNF-α-stimulated T-MSCs (Supplementary Fig. 3A). As a control, in the absence of the IFN-γ/TNF-α stimulation, the splenic cells were distributed rather randomly (Supplementary Fig. 3A). Similar to our previous report with BM-MSCs [39], ICAM-1 and VCAM-1 were greatly induced by IFN-γ/TNF-α stimulation, as measured by real-time PCR and flow cytometry (Supplementary Fig. 3B, C). Moreover, in co-culture of T-MSCs and CD3/CD28-activated T cells, the proliferation of T cells was greatly inhibited (Supplementary Fig. 3D). These results suggest that T-MSCs can produce a large amounts of chemokines and adhesion molecules that are important for immunosuppressive function upon stimulation by proinflammatory cytokines.

BM-MSCs rely on high concentration of local NO to exert immunosuppressive effect on T cells which adhered to MSCs [39]. And *iNos* were upregulated more pronouncedly than *Cox2* and *Pd-l1* under the stimulation of IFN-γ and TNF-α (Fig. 3A). Therefore, we next focused on *iNos* as a mediator of the immunosuppressive effect of T-MSCs. T-MSCs exhibited a significant increase in iNOS protein level dependent on the concentration and treatment duration of IFN-γ and TNF-α (Fig. 3B, C). This result was also verified in the elevation of total nitrite content in the supernatant (Fig. 3D, E). And iNOS-deficient T-MSCs from *Nos2*^−/−^ mice showed no production of NO, as reflected by nitrate in the cell culture supernatant after T-MSCs were treated with IFN-γ and TNF-α (Fig. 3C, E). We then characterized the immunosuppressive effect of iNOS-deficient T-MSCs and their wild-type controls by co-culturing T-MSCs with splenocytes. In co-cultures of wild-type T-MSCs and splenocytes plus anti-CD3, splenocyte proliferation was dose-dependently inhibited by T-MSCs (Fig. 3F, G). The suppressive effect of T-MSCs, however, was significantly decreased in the presence of N^G^-monomethyl-L-arginine (L-NMMA) (Fig. 3G). Furthermore, iNOS-deficient (*Nos2*^-/-^) T-MSCs were compromised in their ability to inhibit the proliferation of splenocytes or CD3^+^ T cells at the ratios of 1:60 and 1:20 (Fig. 3G, H).

**Fig. 4:**
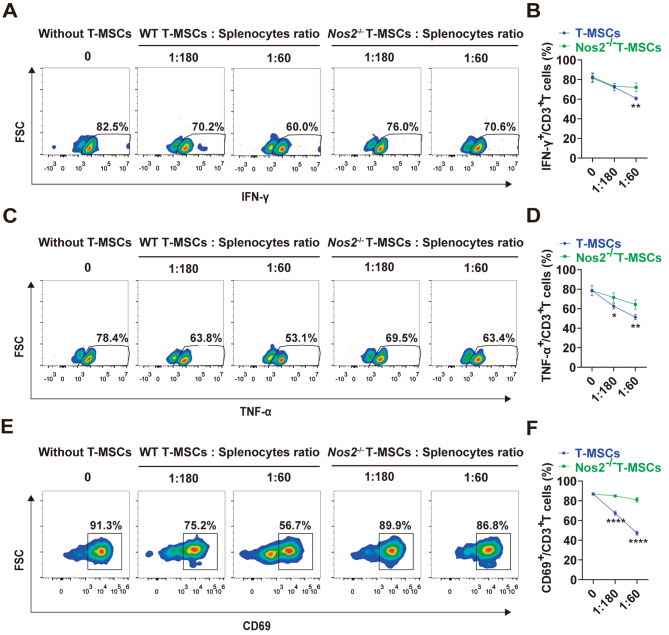
The immunosuppressive effect of T-MSCs on T cell function is partially dependent on NO. **(A)** T-MSCs from *Nos2*^−/−^ or wild-type C57BL/6 mice were cocultured with CD3/CD28-activated C57BL/6 splenocytes, and the pro-inflammatory cytokine IFN-γ secretion of T cells was evaluated by flow cytometry. Representative plots of IFN-γ production by T cells at different ratios. n = 4. **(B)** Bar graphs showed the inhibition rate of IFN-γ-producing T cells after coculture with T-MSCs and *Nos2*^−/−^ T-MSCs. **(C)** T-MSCs from *Nos2*^−/−^ or wild-type C57BL/6 mice were cocultured with CD3/CD28-activated C57BL/6 splenocytes, and the pro-inflammatory cytokine TNF-α secretion of T cells was evaluated by flow cytometry. Representative plots of IFN-γ production by T cells at different ratios. n = 4. **(D)** Bar graphs showed the inhibition rate of TNF-α-producing T cells after coculture with T-MSCs and *Nos2*^−/−^ T-MSCs. **(E)** T-MSCs from *Nos2*^−/−^ or wild-type C57BL/6 mice were cocultured with fresh C57BL/6 splenocytes plus PMA (100 ng/mL), and the percentage of CD69 was evaluated by flow cytometry. Representative plots of CD69 expression by T cells at different ratios. n = 4. **(F)** Bar graphs showed the inhibition rate of T cells activation after coculture with T-MSCs and *Nos2*^−/−^ T-MSCs. Data are represented as mean ± SEM. ns, no significant difference, *p < 0.05, **p < 0.01, ****p < 0.0001

We next determined the effects of T-MSCs on the production of proinflammatory cytokines by CD3^+^ T cells. Co-culture of wild-type T-MSCs with splenocytes for 48 h showed that the production of IFN-γ and TNF-α by CD3^+^ T cells was significantly reduced (Fig. 4A-D; Supplementary Fig. 4A, B). In the presence of *Nos2*^-/-^ T-MSCs, however, the reduction of cytokine expression was attenuated (Fig. 4A-D). Furthermore, T cell activation experiment showed that wild-type T-MSCs strongly inhibited T cell activation, manifested as down-regulated expression of activated marker CD69, whereas *Nos2*^-/-^ T-MSCs were less effective (Fig. 4E, F; Supplementary Fig. 4C). Overall, these results strongly suggest that NO produced by proinflammatory cytokine-induced T-MSCs partially mediates the inhibition of T cell proliferation, cytokine production and activation.

### T-MSCs alleviate ConA-induced liver injury

**Fig. 5:**
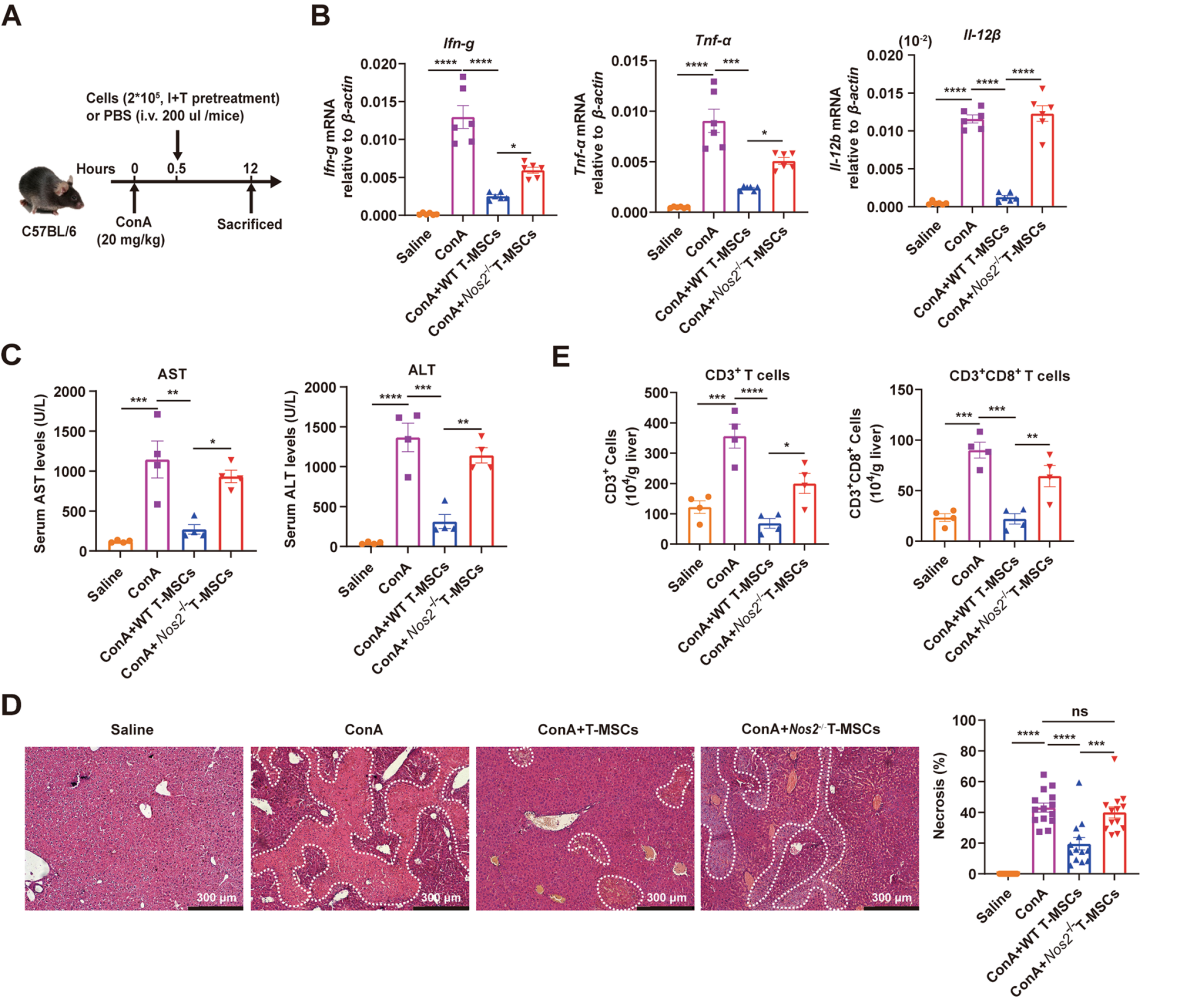
MSCs pretreated with IFN-γ and TNF-α alleviate ConA-induced liver injury in an iNOS-dependent manner. Mice were intravenously injected with ConA (20 mg/kg), 30 min later, IFN-γ and TNF-α-pretreated wild-type or *Nos2*^−/−^ T-MSCs were transfused. After 12 h, serum and livers were sampled. **(A)** Pattern diagram of ConA-induced liver injury in mice prevented by tail vein injection of T-MSCs. **(B)** Expression levels of T-cell specific cytokines were examined by qPCR (n = 6). **(C)** Serum levels of AST and ALT were measured (n = 4). **(D)** Hematoxylin&eosin staining of liver sections at 12 h after ConA administration, and percentages of necrosis were calculated (n = 4). N: necrosis area. Scale bars, 30 μm. **(E)** Absolute numbers of CD3^+^ and CD3^+^CD8^+^ T cells were determined by flow cytometry (n = 4). Data are represented as mean ± SEM. ***p < 0.001, ****p < 0.0001

We next employed the ConA-induced liver injury mouse model to investigate the immunomodulatory effect of T-MSCs in vivo. ConA-induced liver injury is mediated by acute immune responses, in which T cells are identified as the major effector cells [42]. Wild-type T-MSCs or *Nos2*^−/−^ T-MSCs were pretreated with IFN-γ and TNF-α for 24 h, the pretreated MSCs were then intravenously injected into the mice that were adminstered ConA 30 min prior to MSCs injection (Fig. 5A). The results showed that T-MSCs almost completely protected the mice from liver damage, as reflected by a dramatic reduction in serum aspartate aminotransferase (AST) and alanine aminotransferase (ALT) level, in the expression of genes encoding T cell signature cytokines (*Ifn-g*, *Tnf-α*, *Il-12β*), and in centrilobular necrosis (Fig. 5B-D). Analyses of T cells in the liver confirmed that the infiltration of CD3^+^ and CD3^+^CD8^+^ T cells was dramatically decreased in mice administered with T-MSCs (Fig. 5E). However, the amelioration in liver injury was not observed in mice treated with *Nos2*^−/−^ T-MSCs (Fig. 5B-E), indicating that iNOS is a key mediator of immunosuppression by T-MSCs in vivo.

### T-MSCs ameliorate DSS-induced colitis

**Fig. 6:**
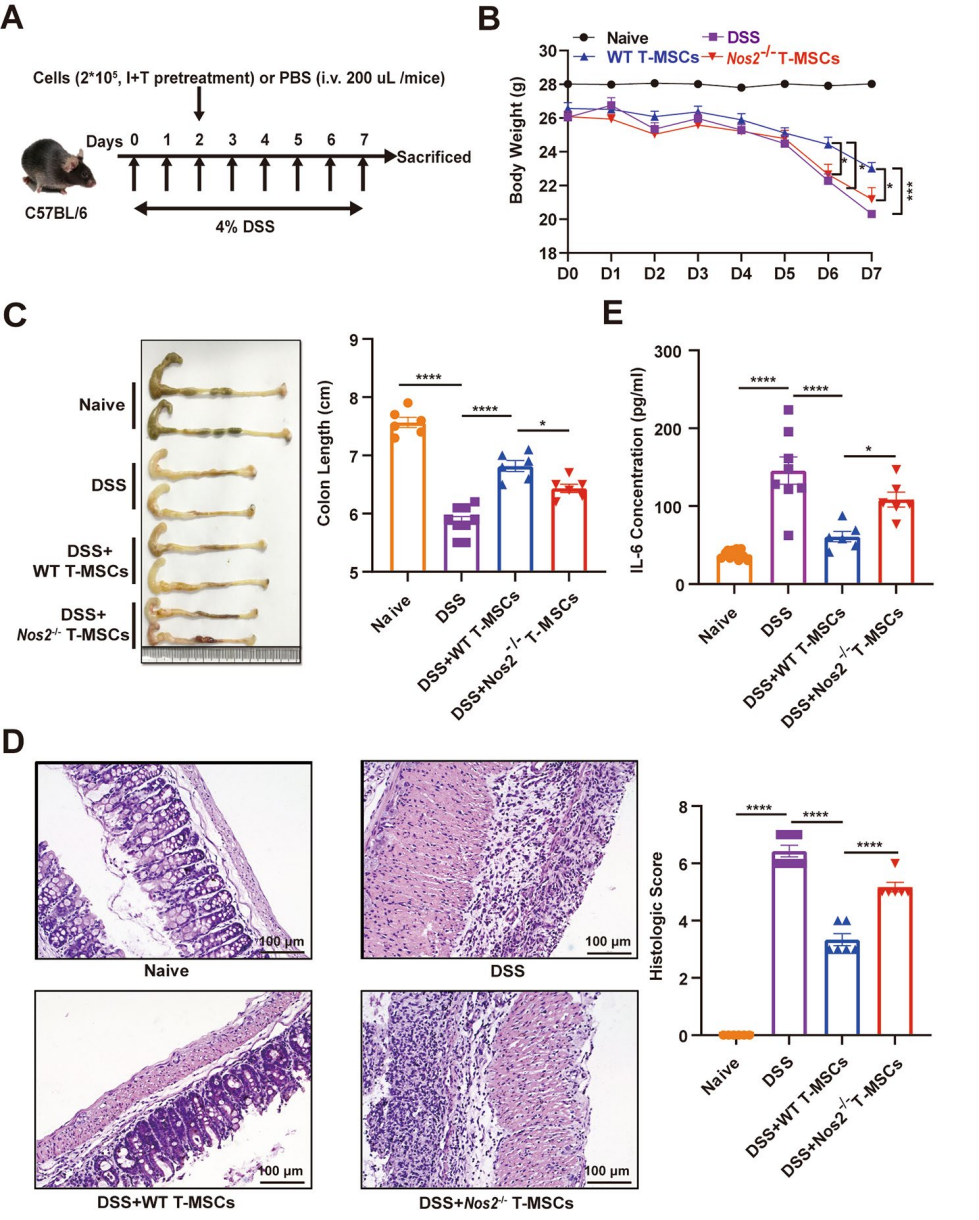
T-MSCs alleviate DSS-induced colitis in a NO production-dependent manner. **(A)** Pattern diagram of DSS-induced colitis in mice prevented by tail vein injection of T-MSCs. **(B, C)** Body weight and colon length of IBD mice treated with phosphate-buffered saline (PBS), T-MSCs, and *Nos2*^−/−^ T-MSCs on day 2 post-IBD induction (**B, C**, n = 6–9). **(D)** Representative H&E-stained colon sections and histological scores of IBD mice treated with PBS, T-MSCs, and *Nos2*^−/−^ T-MSCs on day 2 post-IBD induction (n = 6). Scale bars, 100 μm. **(E)** IL-6 in the serum of IBD mice treated with PBS, T-MSCs, and *Nos2*^−/−^ T-MSCs on day 2 post-IBD induction was assayed by enzyme linked immunosorbent assay (ELISA) (n = 6–8). Data are represented as mean ± SEM. *p < 0.05, ***p < 0.001, ****p < 0.0001

We also tested the effects of T-MSCs on the development of inflammatory tissue damage in a murine model of colitis induced by oral administration of DSS [43]. Similar to previous reports [44, 45], we observed that oral administration of 4% DSS for 7 days induced acute colitis that is characterized by weight loss, reduction of colon length, increased crypt damage, bowel wall thickening and increased infiltration of inflammatory cells (Fig. 6A-D). Moreover, level of IL-6, an important indicator for colitis progression [46], was significantly elevated in colitis (Fig. 6E).

Importantly, intravenous injection with T-MSCs protected mice from colitis-related tissue damage, manifested as reduced loss of body weight and colon length and reduction of IL-6 in serum (Fig. 6B, C, E). Histologically, T-MSCs significantly decreased bowel wall thickness, restored goblet cells and suppressed infiltration of inflammatory cells, thus restoring normal intestinal architecture (Fig. 6D). *Nos2*^−/−^ T-MSCs, on the other hand, barely had beneficial effect on colitis (Fig. 6B-E). These results suggest that T-MSCs can partially alleviate DSS-induced experimental colitis through NO production.

## Discussion

**Fig. 7:**
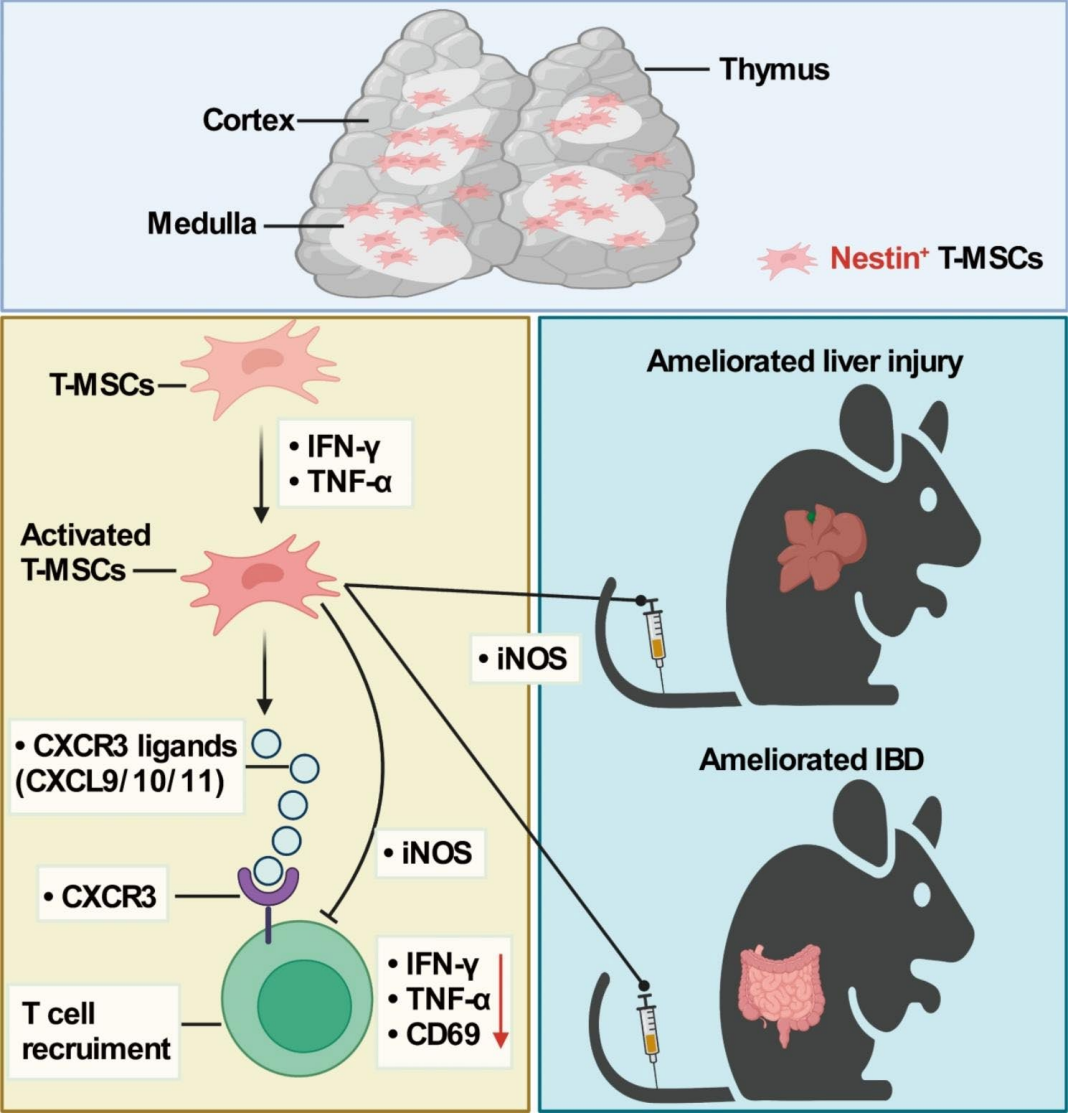
A schematic model of the immunomodulatory mechanism of T-MSCs. T-MSCs stimulated by IFN-γ and TNF-α secrete T cell-specific chemokines and iNOS to recruit T cells and inhibit their proliferation and activation. iNOS mediates the anti-inflammatory effects of T-MSCs, and consequently alleviates IBD and liver injury

In this study, we sought to examine whether thymus-derived MSCs possess immunomodulatory properties and the underlying mechanisms, as well as their spatial distribution in the thymus. We isolated and propagated plastic adherent stromal cells from thymus. Characterization of their spatial distribution in thymus and their immunodulatory function indicate that the T-MSCs may not only provide architectural support, but also possess immunoregulatory property. They are capable to undergo adipogenic and osteogenic differentiation, and express cell surface markers similar to those on BM-MSCs. Moreover, we also identified Nestin^+^ as a marker of T-MSCs and explored their distribution in thymus. The T-MSCs are mainly located in medulla and the cortical-medulla junction, but not in capsule. In addition, when stimulated by INF-γ and TNF-α, Nestin^+^ T-MSCs can secrete immunomodulation-related factors, among which NO is a key effector molecule for MSCs to exert immunosuppressive ability. T-MSCs stimulated by INF-γ and TNF-α can inhibit T cell proliferation and function in vitro and ameliorate acute liver injury and IBD in an iNOS-dependent manner (Fig. 7).

Studies on MSCs in thymus have mainly been conducted in vitro. T-MSCs were found to have the capacity to differentiate into adipocytes and osteoblasts and inhibit activated T lymphocytes-mediated immune responses [47]. However, T-MSCs were not observed to block ConA-stimulated T cell proliferation in another study [41]. We here demonstrated that T-MSCs stimulated by IFN-γ/TNF-α highly expressed genes encoding T cell-specific chemokines *Cxcl9*, *Cxcl10*, *Cxcl11* and immunosuppression-related effector molecules *iNos*, *Cox2* and *Pd-l1*. Moreover, measurements of proliferation, cytokine production and activation of T lymphocytes indicated potent inhibitory effect of T-MSCs on T cells. NO is known to be a key mediator of the immunomodulatory function of BM-MSCs stimulated by inflammatory factors [9]. Here, we demonstrated that the inhibitory effect of T-MSCs on T cells was also partially dependent on NO. When applied to mice with inflammatory diseases, such as acute liver injury and IBD, T-MSCs significantly controlled the inflammatory response, and the therapeutic effect was also dependent on NO, which suggested that NO may be the crucial effector molecule for the immunosuppressive function of MSCs derived from different organs. Beside NO, whether other effector molecules, such as PD-L1 and TGF-β, also play a crucial role remains to be further investigated.

Thymic mesenchyme is heterogeneous and consists of distinct subpopulations, including capsular CD26^+^gp38^+^ cells and medullary CD26^−^gp38^+^ cells [22, 48]. The CD26^−^gp38^+^ cells highly express genes encoding key extracellular matrix components, Wnt signaling molecules and growth factors, which are known to be pivotal for thymic stromal interactions with thymocytes [22, 48, 49]. Medullary fibroblasts (mFbs) are less abundant and the level of gp38 expression is reduced in mice lacking single positive (SP) thymocytes (Tcrα^−/−^), like in newborn thymuses, suggesting that the interaction with SP thymocytes is required for the generation of mFb [22]. Our results showed Nestin^+^ T-MSCs expressed major histocompatibility complex class I (MHC-I) but not MHC-II (data not shown), indicating that these cells do not function as professional antigen-presenting cells. Nestin expression also identifies neural stem cells. Our observation that Nestin is expressed in medullary T-MSCs is consistent with previous reports that T-MSCs are derived from neural crest during thymic development [22, 48]. Unlike CD26 and gp38 that also marks capsular fibroblasts, Nestin is not expressed in capsule and may serve as a more unique marker for medullary mesenchymal cells. Whether the Nestin^+^ T-MSCs are involved in the negative selection of thymocytes to induce CD8^+^ T cell tolerance remains to be determined.

Lymphoprogenitor cells from bone marrow mainly enter thymus via the cortical-medulla junction [50], where Nestin^+^ cells are also localized. BM-MSCs are known to be critical for supporting hematopoietic stem cells [2, 3] and the transcriptomic profile of the thymic MCs suggests that T-MSCs may play multiple roles in T cell development [33]. It is possible that Nestin^+^ cells may participate in the formation of a supportive niche for lymphoprogenitor cells niche or in promoting the clearance of apoptotic cells by macrophages or interacting with epitheliums [48]. Thymus, one of the earliest organs to enter atrophy, exhibits increased inflammation during involution [51, 52]. Whether or not the elevation of inflammatory factors may drive T-MSCs to exert immunosuppressive effect in thymus deserves further study.

## Conclusions

In conclusion, T-MSCs exhibited characteristics of mesenchymal stromal cells and immunomodulatory properties similar to BM-MSCs. Meanwhile, T-MSCs exert potent immunomodulatory effect through iNOS in acute liver injury and IBD mouse models. T-MSCs can be identified by their expression of nestin and are primarily localized in the medulla and medulla-cortex junction. Our findings expand the sources of MSCs and have implications in understanding the physiological function of resident mesenchymal stem cells in the thymus.

## Methods

### Animals

Homozygous *Nos2*^tm1Lau^ mutant (also known as iNOS-) mice (Stock No: 002596) of C57BL/6 genetic background were obtained from the Jackson Laboratory. C57BL/6 wild-type mice were purchased from the Shanghai SLAC Laboratory Animal Co. Ltd. (Shanghai, China). B6.Cg-Tg(*Nes-cre*)1Kln/J (Nestin-cre) mice (Stock No: 003771), B6.129 × 1-Twist2^tm1.1(cre)Dor^/J (Dermo1-cre) mice (Stock No: 008712), B6.129 (Cg)-Lepr^tm2(*cre*)Rck^/J (LeptinR-cre) mice (Stock No: 008320), and B6.Cg-Gt(*ROSA*)26Sor ^tm14(CAG−*tdTomato*)Hze/J^ (tdTomato-flox) reporter mice (Stock No: 007914) were obtained from the Jackson Laboratory. B6/JGpt-Gli1em1Cin(*CreERT2*)/Gpt (Gli1-CreERT2) mice (Stock No: T005677) was obtained from the GemPharmatech Co.,Ltd. Mice were bred in a specific pathogen-free animal facility of Soochow University. The animal protocols for the experiments and care were in full compliance with the care and use of laboratory animals, and the Ethics Committee of Soochow University approved all protocols (SUDA20210916A07).

### Cell cultures

T-MSCs derived from mouse thymus were collected as previously described [53]. Briefly, the thymic lobes were minced into small fragments, then treated for 1 h at 37 °C with an enzymatic mixture containing 0.2 mg/mL DNase type I (Solarbio), 1 mg/mL collagenase IV (Gibco), and 5 mM EDTA (Beyotime) in RPMI. Cells were spun at 300 rcf for 10 min. The pellet was resuspended and cells were then cultured in 25-cm^2^ flasks (Thermo Fisher Nunc) at 5 × 10^6^ nucleated cell concentration in 5 mL of modified Eagle medium containing 20% FBS (Invitrogen), 2.5 ng/mL bFGF (PeproTech), and 33 ng/mL insulin (Sigma-Aldrich). BM-MSCs were collected as previously described [54]. In brief, BM cells were collected by flushing femurs and tibias with complete medium (CM) containing α-MEM supplemented with 10% FBS, 100 U/ml penicillin, 100 µg/ml streptomycin and 2 mM glutamine (all from Invitrogen). Non-adherent cells were removed after 72 h, and then maintained in the culture medium which was replaced every 3 days. T-MSCs and BM-MSCs became more homogeneous after six passages and beared the following markers: CD29^+^ Sca-1^+^ CD44^+^ Nestin^+^ CD140a^low^ CD73^low^ MHCII^−^ CD45^−^ CD11B^−^ CD31^−^ CD34^−^.

### Quantitative real-time PCR

Total RNA was extracted using TRIzol reagent (Sigma-Aldrich), and cDNA synthesis was carried out using PrimeScript™ RT Master Mix (TaKaRa) with random hexamer primers. The generated cDNA was subjected to quantitative real-time PCR using FastStart Universal SYBR Green Master (Applied Biosystems). The primer sequences are listed in the Supplementary information, Table 1.

### Cell differentiation ability in vitro

Adipogenic differentiation: MSCs were cultured in high-glucose DMEM containing 10 µg/mL insulin (Sigma-Aldrich), 0.6 mM indomethacin (Sigma-Aldrich), 100 nM dexamethasone (Sigma-Aldrich), 0.5 mM 3-isobutyl-1 methylxanthine (Sigma-Aldrich), 10% FBS (Invitrogen) and 100 IU/mL penicillin/streptomycin (Invitrogen) for 2 weeks. The medium was changed twice a week. These adipogenic-differentiated cells were analyzed by staining with Oil red O.

Osteogenic differentiation: MSCs were cultured in low-glucose DMEM containing 10% FBS (Invitrogen), 2 mM β-glycerophosphate (Sigma-Aldrich), 10 nM dexamethasone (Sigma-Aldrich), 100 µg/mL ascorbic acid (Sigma-Aldrich), and 100 IU/mL penicillin/streptomycin (Invitrogen) for 3 weeks. The medium was changed twice a week. These osteogenic-differentiated cells were analyzed by staining with Alizarin Red.

### Flow cytometry

Characterization of the surface markers of cells was performed by using flow cytometry. MSCs digested in a dish with 0.25% Trypsin (Biosharp) were suspended in staining buffer (PBS, 1% FBS) and 1 × 10^5^ cells/sample were incubated with fluorescently labeled antibodies (all 1:200) for 30 min at 4 °C. The antibodies used for flow cytometry were as follows: PE anti-mouse CD29 (eBioscience), PE anti-mouse Sca-1 (Biolegend), PE anti-mouse CD140a (eBioscience), PE anti-mouse CD44 (eBioscience), PE anti-mouse CD73 (eBioscience), PE anti-mouse MHC II (eBioscience), PE anti-mouse CD45 (Biolegend), PE anti-mouse CD31 (eBioscience), PE anti-mouse CD34 (Biolegend), PE anti-mouse CD11B (eBioscience), PE anti-mouse ICAM-1 (Biolegend), and BV421 anti-mouse VCAM-1 (BD Pharmingen). For intracellular staining, cells stained with cell surface antibodies (PE anti-mouse CD3 (Biolegend)) were fixed, permeabilized using foxp3/transcription factor concentrate and diluent kit set prior to incubation with antibodies directed at intracellular antigens (APC anti-mouse KI-67 (Biolegend)). Fluorescence intensity was measured by flow cytometry (Cytoflex, Beckman Coulter). Data were analyzed with FlowJo v10 software and Cytexpert software.

### Immunofluorescence

MSCs, cultured in the 12-well plate, were washed in PBS and fixed in 4% PFA for 10 min. After fixation the cells were with 5% normal serum and 0.05% Triton X-100 (Sigma-Aldrich) in PBS for 30 min at room temperature, followed by incubation with antibodies according to the protocol provided by the manufacturer. The cells were examined using a cell imaging microplate detection system-Cytation5 (BioTek) and a laser scanning confocal microscopy (Leica Biosystems). Primary antibodies (1:300) were applied as follows: Rat monoclonal anti-Nestin (Abcam ab81462), Rabbit monoclonal anti-Alpha smooth muscle actin (Abcam ab124964) and Rabbit monoclonal anti-to Cytokeratin 14 (Abcam ab181595). The following secondary antibodies (1:1000) were used: Alexa Fluor 488 Goat anti-Rat IgG (Abcam ab150157), Alexa Fluor 488 Goat anti-Rabbit IgG (Abcam ab150077). Nuclei were counterstained with Hoechst 33,342 (1:1000) (Invitrogen).

### Proliferation assays

Splenocytes (5 × 10^5^) were cultured in 500 µL of complete medium in 48-well plate for the indicated times. De novo cell proliferation was analyzed in cells stained with carboxyfluorescein diacetate succinimidyl ester (CFSE): freshly isolated splenocytes (10^7^/mL in PBS) were labeled with 5 µM CFSE for 8 min at 37 °C. After washing twice in PBS with 2% FBS, CSFE-labeled T cells were cultured in a 48-well plate, pre-coated with anti-CD3, and soluble anti-CD28 was added to the medium to induce T-cell proliferation either with or without MSCs at the indicated ratios. Three days later, cells were collected and fluorescence intensity analyzed by flow cytometry.

### Cytokine assays

CD3/CD28-activated splenocytes were cultured with or without MSCs for 2 days. During the last 6 h of incubation, ionomycin (500 ng/mL), phorbol-12-myristate-13-acetate (PMA, 50 ng/mL), and brefeldin A (BFA, 10 µg/mL) were added to the culture system (all from Sigma-Aldrich Aldrich). IFN-γ and TNF-α were analyzed by flow cytometry.

### T cell activation

Splenocytes treated with PMA (100 ng/mL) were cultured with or without MSCs cells for 24 h. The expression of CD69 was analyzed by flow cytometry.

### IL-6 ELISA

For IL-6 analysis, mouse serum was collected by centrifugation at 7000 rpm for 20 min at 26 °C. The content of IL-6 in serum was measured by Mouse IL-6 ELISA kit (Beyotime) according to protocol provided by the manufacturer. In brief, the standard products or samples were added to the pre-coated enzyme plate, and double antibody sandwich ELISA was used to enable the target protein to be colored by the tetramethylbenzidine (TMB) solution. Finally, the absorbance value at 450 nm was detected by the enzyme-labeled instrument. The IL-6 protein concentration in the sample can be calculated by comparing with the absorbance value of the standard products.

### Measurement of NO

The levels of NO in the medium of cultured MSCs were measured as nitrite (NO^2−^) using a Griess reagent kit (Sigma-Aldrich) according to the manufacturer’s instruction. Briefly, 50 µL of culture supernatant was gently mixed with 50 µL of Griess reagent (modified) and incubated in a shaker at room temperature for 15 min. The absorbance at 540 nm was measured in a microplate reader (Cytation5, BioTek).

### ConA-induced liver injury in mice

C57BL/6 mice (8–10 week old) were intravenously injected with ConA in saline at 20 mg/kg to induce liver injury. MSCs (2 × 10^5^) were treated with IFN-γ and TNF-α (10 ng/mL for each cytokine) for 24 h, and then, these cells (2 × 10^5^ cells in 200 µL PBS) or PBS, respectively, were administered intravenously into mice that have been treated with ConA for 30 min. Mice were euthanized and serum and liver tissues were sampled after another 11.5 h. Serum AST and ALT activity were determined by HITACHI AUTOMATIC ANALYZER (Japan). 4% Paraformaldehyde-fixed liver histological sections were stained with hematoxylin & eosin.

### DSS-induced colitis and experimental therapies

Colitis was induced by 4% Dextran Sulfate Sodium (DSS) (MP Biomedicals) in drinking water ad libitum for 7 days. On day 2, MSCs pretreated with IFN-γ and TNF-α for 24 h were harvested, and then, these cells (2 × 10^5^ cells in 200 µL PBS) or PBS, respectively, were administered intravenously into mice. Body weight of mice was recorded every day. Mice were euthanized on day 7 for detection of serum IL-6 levels and tissue histological analysis.

### Histological analysis of colons

Colon tissues were fixed in 4% paraformaldehyde overnight at room temperature. Colons were then dehydrated through sequentially alcohol concentration gradient and treated with xylene before embedded in paraffin. The samples were then cut into 4-µm-thick sections, which were performed using standard Hematoxylin&Eosin (H&E). The severity of colitis was evaluated by scoring the damage of crypt (grades, 0–3: 0, none; 1, loss of goblet cells; 2, only surface epithelium intact; 3, loss of entire crypt and epithelium), the extent of bowel wall thickening (grades, 0–3: 0, none; 1, mucosa; 2, mucosa and submucosa; 3, transmural), and the infiltration of inflammatory cells (grades, 0–2: 0, none; 1, mild to moderate; 2, severe).

### Statistical analysis

All data are presented as the mean ± standard error of the mean (SEM). The GraphPad Prism 9 software was used for the statistical analyses. For two-group comparison, One-tailed paired t test was performed. And statistical significance was assessed by one-way analysis of variance test when more than two groups were compared. ns, not significant; p < 0.05, significant.

### Electronic supplementary material

Below is the link to the electronic supplementary material.

**Additional File 1: Supplementary Table 1**

**Additional File 2: Supplementary Fig. 1**. The gene expression profiles of T-MSCs and BM-MSCs. The heatmap of gene expression in thymocytes, T-MSCs, BM-MSCs and ADSCs were obtained by real-time PCR analysis.

**Additional File 3: Supplementary Fig. 2**. Expression of immunosuppressive factors in BM-MSCs. (A-F) The expression levels of Cxcl9, Cxcl10, Cxcl11, iNos, Cox2 and Pd-l1 were measured by real-time PCR at 12 h, 24 h and 48 h after BM-MSCs were stimulated with 20 ng/mL IFN-γ and TNF-α alone or in combination. Data are represented as mean ± SEM. *P < 0.05, **P < 0.01, ***P < 0.001, ****P < 0.0001.

**Additional File 4: Supplementary Fig. 3**. Upregulation of ICAM-1 and VCAM-1 in T-MSCs under the stimulation of proinflammatory factors. (A) T-MSCs were cocultured with splenocytes (1:10 ratio) with or without IFN-γ and TNF-α (20 ng/mL each) for 12 h, and the extent of cell aggregation was examined microscopically. Scale bars, 100 µm. (B, C) T-MSCs derived from C57BL/6 mice were treated with IFN-γ/TNF-α (20 ng/mL each) for 24 h. (B) The expression of Icam-1 and Vcam-1 in treated and control T-MSCs was analyzed by real-time PCR. (C) The expression of ICAM-1 and VCAM-1 was analyzed by flow cytometry. (D) Fresh C57BL/6 splenocytes were stimulated with or without anti-CD3/CD28 (1 µg/mL) and cultured in the presence or absence of T-MSCs derived from C57BL/6 mice at a 20:1 ratio (splenocytes:T-MSCs). The cells were examined microscopically after 48 h. Scale bars, 100 µm. Representative of 3 independent experiments. Data are represented as mean ± SEM. ns, no significant difference, ***p < 0.001, ****p < 0.0001.

**Additional File 5: Supplementary Fig. 4**. Evaluation of IFN-γ, TNF-α and CD69 expression by flow cytometry. (A) C57BL/6 splenocytes were stimulated with or without anti-CD3/CD28 (1 µg/mL each), and the pro-inflammatory cytokine IFN-γ secretion of T cells was evaluated by flow cytometry. Representative plots of IFN-γ production by T cells at different ratios. n = 4. (B) C57BL/6 splenocytes were stimulated with or without anti-CD3/CD28 (1 µg/mL each), and the pro-inflammatory cytokine TNF-α secretion of T cells was evaluated by flow cytometry. Representative plots of TNF-α production by T cells at different ratios. n = 4. (C) C57BL/6 splenocytes were stimulated with or without PMA (100 ng/mL), and the percentage of CD69 was evaluated by flow cytometry. Representative plots of CD69 expression by T cells at different ratios. n = 4.

## Data Availability

The datasets used and/or analysed during the current study are available from the corresponding author on reasonable request.

## Abbreviations

BM-MSCs: Bone marrow-derived MSCs
ConA: Concanavalin A
*Cox2*: Cyclooxygenase 2
DSS: Dextran sulfate sodium
EDTA: Ethylene diamine tetraacetic acid
FBS: Fetal bovine serum
IBD: Inflammatory bowel disease
IFN-γ: Interferon-γ
IL-6: Interleukin-6
*Il-12β*: Interleukin-12
iNOS: Inducible nitric oxide synthase
L-NMMA: NG-monomethyl-L-arginine
MSCs: Mesenchymal stem/stromal cells
NO: Nitric oxide
PBS: Phosphate-bufered saline
*Pd-l1*: Programmed death ligand 1
PFA: Paraformaldehyde
T-MSCs: Thymus-derived MSCs
TNF-α: Tumor necrosis factor-α
