# Supplementary figures and images for: Nitric oxide-dependent immunosuppressive function of thymus-derived mesenchymal stromal/stem cells
Source: Biol Direct. 2023 Sep 18;18:59. doi: 10.1186/s13062-023-00415-4 (PMC10506207; doi:10.1186/s13062-023-00415-4)

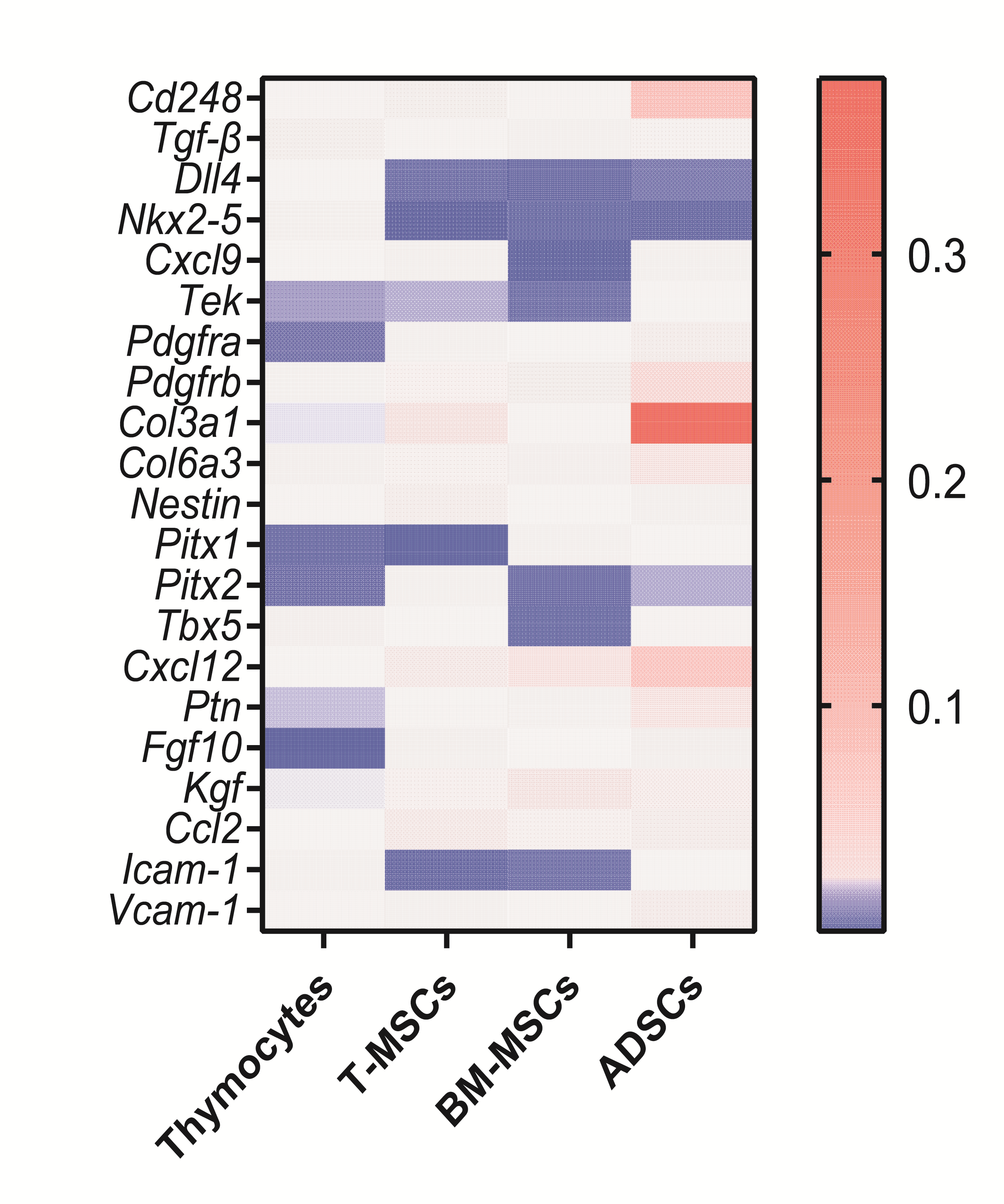

Supplement: Supplementary file 2 — Additional File 2: Supplementary Fig. 1. The gene expression profiles of T-MSCs and BM-MSCs. The heatmap of gene expression in thymocytes, T-MSCs, BM-MSCs and ADSCs were obtained by real-time PCR analysis. [file 13062_2023_415_MOESM2_ESM.png]

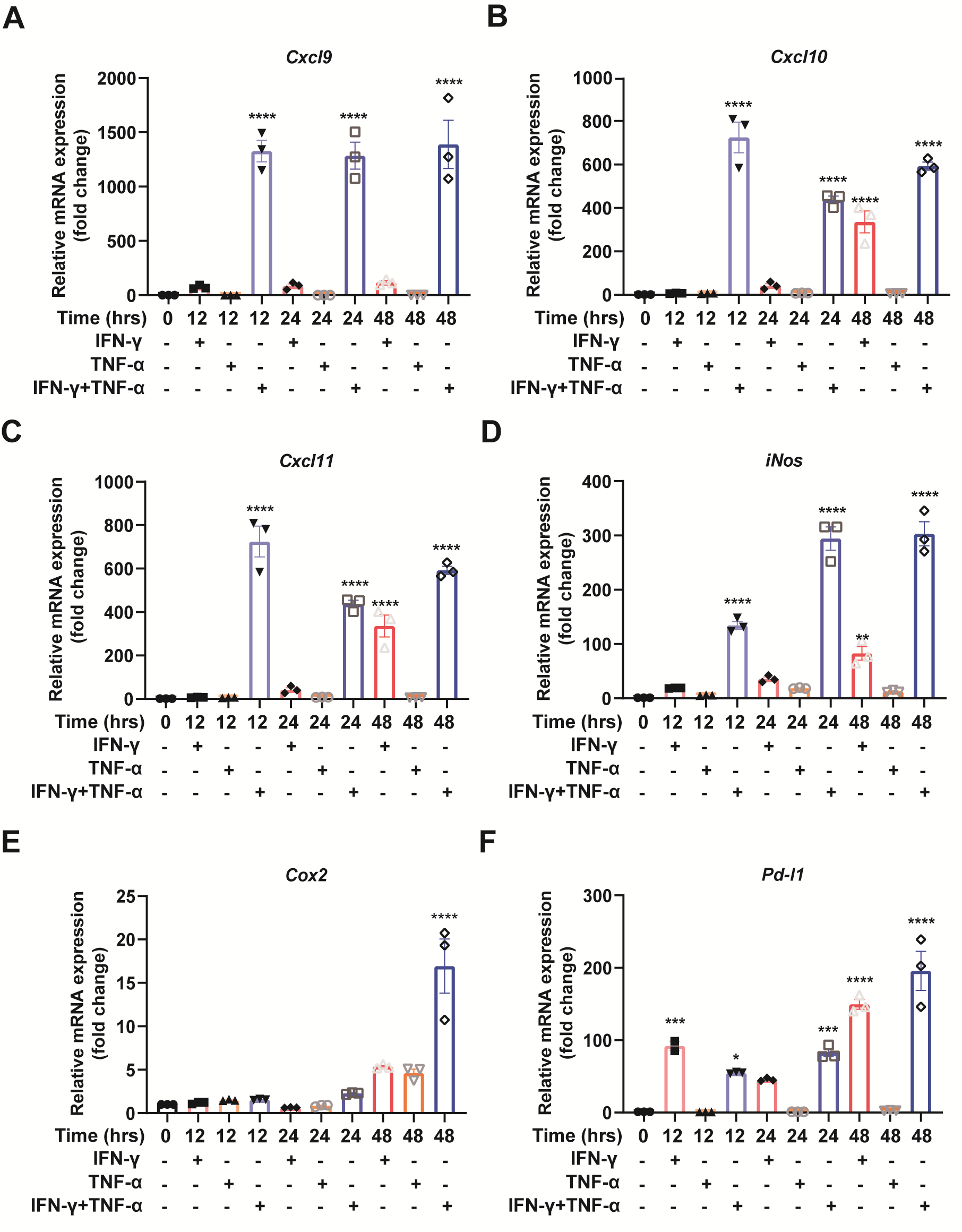

Supplement: Supplementary file 3 — Additional File 3: Supplementary Fig. 2. Expression of immunosuppressive factors in BM-MSCs. (A-F) The expression levels of Cxcl9, Cxcl10, Cxcl11, iNos, Cox2 and Pd-l1 were measured by real-time PCR at 12 h, 24 h and 48 h after BM-MSCs were stimulated with 20 ng/mL IFN-γ and TNF-α alone or in combination. Data are represented as mean ± SEM. *P < 0.05, **P < 0.01, ***P < 0.001, ****P < 0.0001. [file 13062_2023_415_MOESM3_ESM.png]

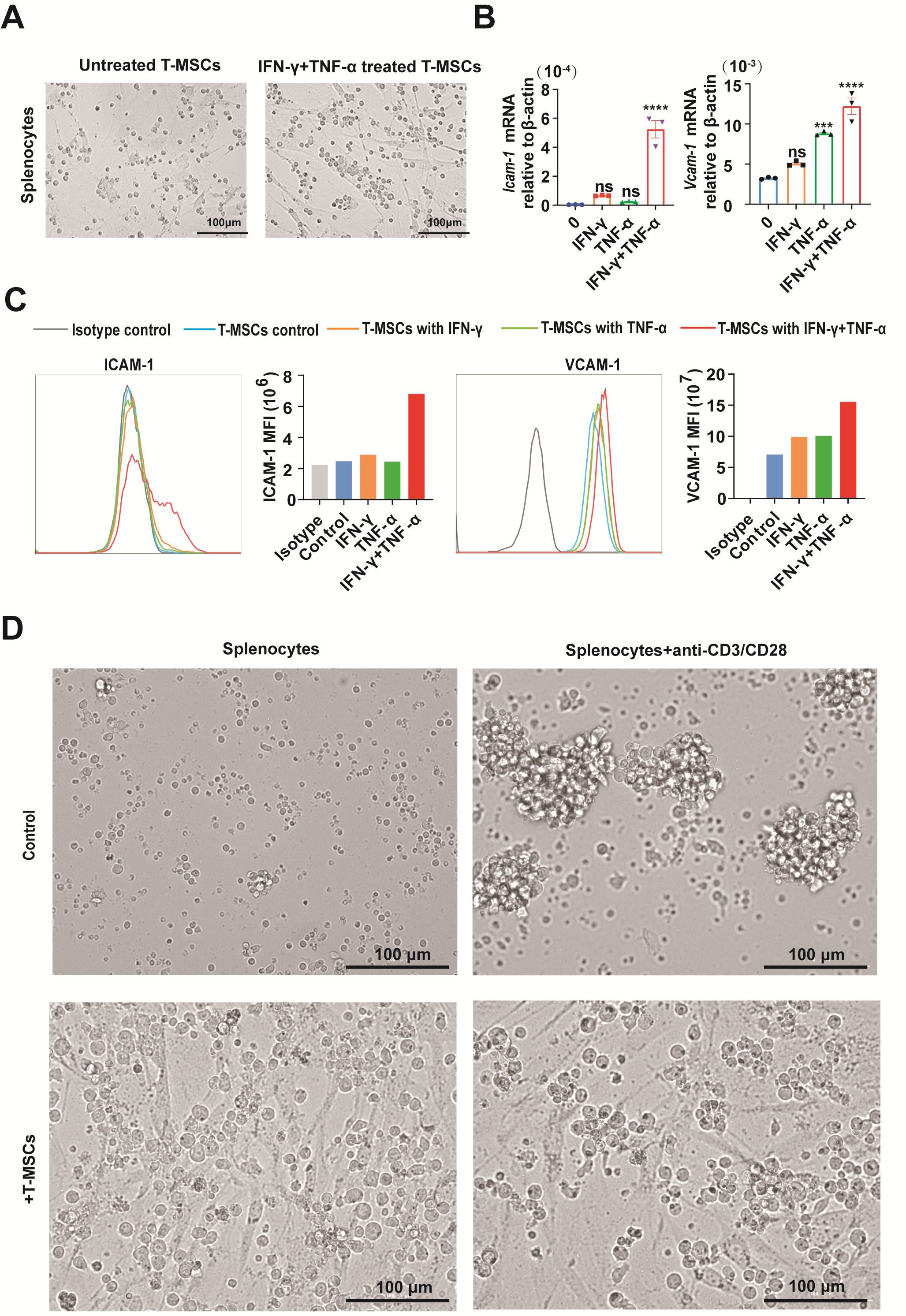

Supplement: Supplementary file 4 — Additional File 4: Supplementary Fig. 3. Upregulation of ICAM-1 and VCAM-1 in T-MSCs under the stimulation of proinflammatory factors. (A) T-MSCs were cocultured with splenocytes (1:10 ratio) with or without IFN-γ and TNF-α (20 ng/mL each) for 12 h, and the extent of cell aggregation was examined microscopically. Scale bars, 100 µm. (B, C) T-MSCs derived from C57BL/6 mice were treated with IFN-γ/TNF-α (20 ng/mL each) for 24 h. (B) The expression of Icam-1 and Vcam-1 in treated and control T-MSCs was analyzed by real-time PCR. (C) The expression of ICAM-1 and VCAM-1 was analyzed by flow cytometry. (D) Fresh C57BL/6 splenocytes were stimulated with or without anti-CD3/CD28 (1 µg/mL) and cultured in the presence or absence of T-MSCs derived from C57BL/6 mice at a 20:1 ratio (splenocytes:T-MSCs). The cells were examined microscopically after 48 h. Scale bars, 100 µm. Representative of 3 independent experiments. Data are represented as mean ± SEM. ns, no significant difference, ***p < 0.001, ****p < 0.0001. [file 13062_2023_415_MOESM4_ESM.png]

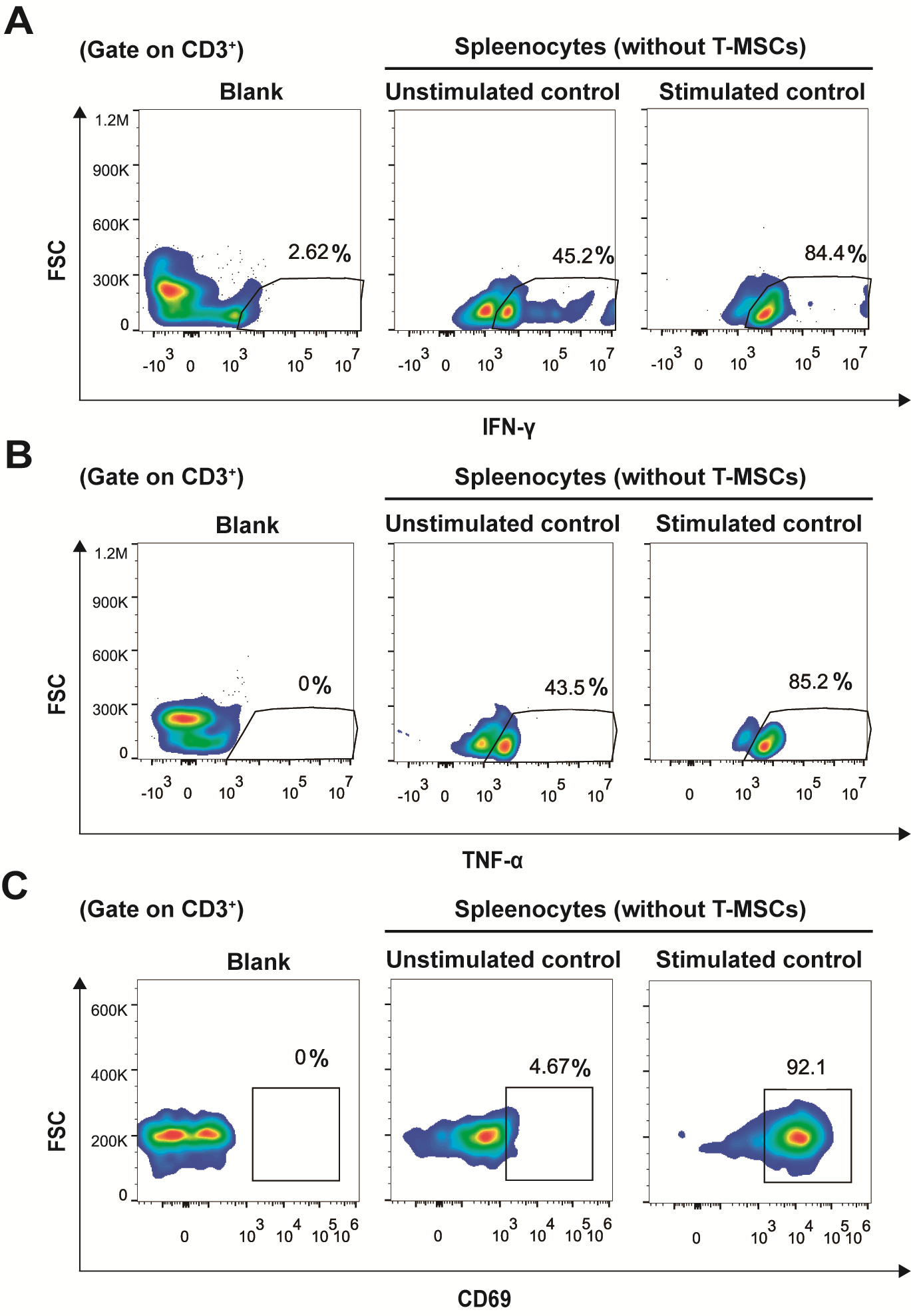

Supplement: Supplementary file 5 — Additional File 5: Supplementary Fig. 4. Evaluation of IFN-γ, TNF-α and CD69 expression by flow cytometry. (A) C57BL/6 splenocytes were stimulated with or without anti-CD3/CD28 (1 µg/mL each), and the pro-inflammatory cytokine IFN-γ secretion of T cells was evaluated by flow cytometry. Representative plots of IFN-γ production by T cells at different ratios. n = 4. (B) C57BL/6 splenocytes were stimulated with or without anti-CD3/CD28 (1 µg/mL each), and the pro-inflammatory cytokine TNF-α secretion of T cells was evaluated by flow cytometry. Representative plots of TNF-α production by T cells at different ratios. n = 4. (C) C57BL/6 splenocytes were stimulated with or without PMA (100 ng/mL), and the percentage of CD69 was evaluated by flow cytometry. Representative plots of CD69 expression by T cells at different ratios. n = 4. [file 13062_2023_415_MOESM5_ESM.png]
